# Exogenous Carbon Monoxide Decreases Sepsis-Induced Acute Kidney Injury and Inhibits NLRP3 Inflammasome Activation in Rats

**DOI:** 10.3390/ijms160920595

**Published:** 2015-08-31

**Authors:** Peng Wang, Jian Huang, Yi Li, Ruiming Chang, Haidong Wu, Jiali Lin, Zitong Huang

**Affiliations:** 1 Department of Emergency Medicine, Sun Yat-sen Memorial Hospital of Sun Yat-sen University, Guangzhou 510120, China; E-Mails: pengwangsysu@foxmail.com (P.W.); doctorli113@163.com (Y.L.); rubby866@126.com (R.C.); wywhd@163.com (H.W.); cheatl@163.com (J.L.); 2 Institute of Cardiopulmonary Cerebral Resuscitation, Sun Yat-sen University, Guangzhou 510120, China; 3 Department of Nephrology, the Fourth Affiliated Hospital of Guangzhou Medical University, Guangzhou 510120, China; E-Mail: hj510182@126.com

**Keywords:** acute kidney injury, carbon monoxide, NLRP3 inflammasome, sepsis

## Abstract

Carbon monoxide (CO) has shown various physiological effects including anti-inflammatory activity in several diseases, whereas the therapeutic efficacy of CO on sepsis-induced acute kidney injury (AKI) has not been reported as of yet. The purpose of the present study was to explore the effects of exogenous CO on sepsis-induced AKI and nucleotide-binding domain-like receptor protein 3 (NLRP3) inflammasome activation in rats. Male rats were subjected to cecal ligation and puncture (CLP) to induce sepsis and AKI. Exogenous CO delivered from CO-releasing molecule 2 (CORM-2) was used intraperitoneally as intervention after CLP surgery. Therapeutic effects of CORM-2 on sepsis-induced AKI were assessed by measuring serum creatinine (Scr) and blood urea nitrogen (BUN), kidney histology scores, apoptotic cell scores, oxidative stress, levels of cytokines TNF-α and IL-1β, and NLRP3 inflammasome expression. CORM-2 treatment protected against the sepsis-induced AKI as evidenced by reducing serum Scr/BUN levels, apoptotic cells scores, increasing survival rates, and decreasing renal histology scores. Furthermore, treatment with CORM-2 significantly reduced TNF-α and IL-1β levels and oxidative stress. Moreover, CORM-2 treatment significantly decreased NLRP3 inflammasome protein expressions. Our study provided evidence that CORM-2 treatment protected against sepsis-induced AKI and inhibited NLRP3 inflammasome activation, and suggested that CORM-2 could be a potential therapeutic candidate for treating sepsis-induced AKI.

## 1. Introduction

Sepsis is a severe clinical syndrome encountered and triggered in patients with infection, which is associated with significant mortality and morbidity. Sepsis may lead to multiple organ failure, and the kidney is a commonly affected organ during sepsis [1,2]. It is well established that septic patients are particularly at risk of developing acute kidney injury (AKI), and carry a high risk of mortality. The pathophysiology of AKI in sepsis is complex and multi-factorial and includes pro-inflammatory cytokines, chemokines, and reactive oxygen species (ROS) [1,2]. Despite extensive research and progress in several fields of AKI, the appropriate therapeutic interventions are still limited and mortality of septic AKI remains unacceptably high.

The nucleotide-binding domain-like receptor protein 3 (NLRP3) inflammasome is a cytosolic protein complex composed of NLRP3, the adaptor protein ASC, and caspase-1 [3]. The activation of NLRP3 inflammasome promotes the maturation and release of several pro-inflammatory cytokines including interleukin-1β (IL-1β) and IL-18, so it plays critical roles in the initiation of inflammation and the development of immune responses [4]. The NLRP3 inflammasome can be activated by a broad spectrum of stimulating factors such as bacteria, virus, fungi, and components of dying cells [4]. The mechanisms by which these structurally distinct factors trigger NLRP3 inflammasome activation are currently unclear. However, one of the models proposes that NLRP3 can be activated by a common pathway of ROS [5,6]. Several studies have shown that dysregulated NLRP3 inflammasome activation participates in the pathogenesis of sepsis and AKI [7,8,9]. As the pro-inflammatory effect of the NLRP3 inflammasome is deleterious, the discovery of effective and specific drugs that alter NLRP3 inflammasome function has the potential to improve the symptoms of sepsis and AKI.

Carbon monoxide (CO) is best known as a toxic gas which binds to heme and thereby forming carboxyhemoglobin and severely impairing the respiratory system. However, endogenous CO is a by-product of inducible heme oxygenase, and exerts various physiological effects including anti-oxidant and anti-inflammatory activity [10]. Recently, transition metal carbonyls have been identified as novel CO-releasing molecules (CORMs) and as therapeutic agents aimed at delivering controlled amounts of CO to tissues and organs [11,12]. A metal carbonyl complex tricarbonyldichlororuthenium(II) dimer (CORM-2) or CORM-3 has shown biological activities in reducing hypertension, attenuating sepsis-induced cardiac dysfunction and improving outcome of ischemia reperfusion without significant toxicity [11,13,14,15,16]. However, little is known about whether or not CORM-2 released CO can protect against sepsis-induced AKI in rats. Therefore, the aim of the present study was to investigate the effects of CORM-2 on sepsis-induced AKI and NLRP3 inflammasome activation in rats.

## 2. Results

### 2.1. Carbon Monoxide Decreased Sepsis-Induced Acute Kidney Injury and Improved Survival of the Rats

**Figure 1:**
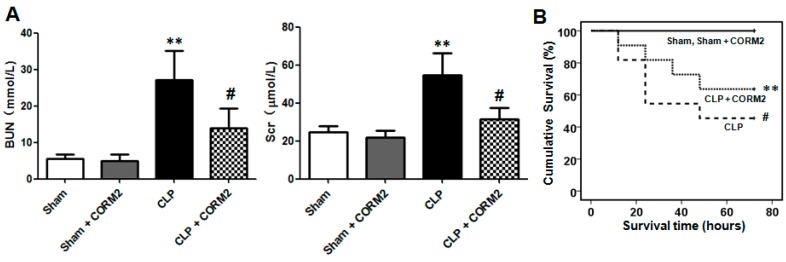
Effects of carbon monoxide on acute kidney injury and survival of septic rats. (**A**) Carbon monoxide significantly decreased blood urea nitrogen (BUN) and serum creatinine (Scr) levels of the cecal ligation and puncture (CLP)-induced septic rats. CORM-2 had no effects on BUN and Scr to normal rats (Sham). Results are presented as mean ± SD, *n* = 6 rats per group. ** *p* < 0.01 *vs.* sham group and **^#^** *p* < 0.05 *vs.* CLP group; (**B**) Carbon monoxide significantly increased the survival of the CLP-induced septic rats. CORM-2 had no effect on survival to normal rats (Sham). The survival rate was determined by Kaplan-Meier method and compared by log-rank test. *n* = 11 rats per group. ** *p* < 0.01 *vs.* sham group and **^#^** *p* < 0.05 *vs.* CLP group. Sham indicates no CLP surgery control group; Sham + CORM-2 indicates sham plus CORM-2 treatment group; CLP indicates CLP surgery group; CLP + CORM-2 indicates CLP plus CORM-2 treatment group.

AKI is a frequent and serious complication induced by sepsis. Blood urea nitrogen (BUN) and serum creatinine (Scr) are frequently-used biomarkers in early stages of the development of AKI [17,18]. To detect the renal function of cecal ligation and perforation (CLP) induced sepsis, Scr and BUN levels were measured at 12 h after CLP surgery. The results showed that the Scr and BUN levels were significantly increased after CLP surgery (Figure 1A), which indicated that the renal function was decreased in CLP-induced septic rats.

CORM-2 was used to study the effects on sepsis induced AKI in rats. The results showed that CORM-2 treatment significantly decreased the Scr and BUN levels in CLP-induced septic rats (Figure 1A). Sepsis with AKI had a high mortality, and about 50% of the rats were dead within three days. However, CORM-2 treatment significantly increased the three-day survival time of the rats (Figure 1B). Taken together, these data suggest that CORM-2 decrease sepsis-induced acute kidney injury and improved survival of the rats.

### 2.2. Carbon Monoxide Attenuated Renal Pathological Injury in Septic Rats

**Figure 2:**
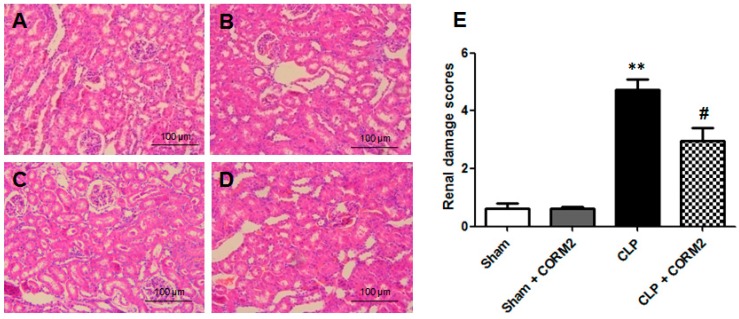
Carbon monoxide decreased renal injury by histopathological examination in septic rats. (**A**–**D**) The renal tissues were stained with hematoxylin and eosin (HE) and evaluated histopathological changes. (**A**) Sham group; (**B**) Sham + CORM-2 group; (**C**) CLP group; (**D**) CLP + CORM-2 group. Scale bar = 100 μm; (**E**) Semi-quantitative renal injury analysis was evaluated in HE-stained renal tissue sections. CLP-induced sepsis leads to histopathological injury in renal tissues and CORM-2 treatment decreased injury in renal tissues. CORM-2 had no effect on renal tissues histopathological changes to normal rats (Sham). Results are presented as mean ± standard deviation (SD), *n* = 6 rats per group. ** *p* < 0.01 *vs.* sham group and **^#^** *p* < 0.05 *vs.* CLP group.

To examine the protective effect of CORM-2 on sepsis induced AKI in rats, the renal histopathologic changes were observed at 24 h after CLP surgery. As shown in Figure 2A–D, renal sections of normal sham group and sham plus CORM-2 group showed normal tubular architecture and cellular structure. Renal sections obtained from CLP group showed significant pathologic changes, including loss of brush border, bleb formation, and tubular luminal debris or obstruction. However, these pathological changes were significantly attenuated by CORM-2 treatment. The histopathologic morphologies were scored with a semiquantitative scale evaluating changes found in AKI. As shown in Figure 2E, CORM-2 treatment significantly decreased mean histologic scores of the CLP- induced AKI in rats (*p* < 0.05).

### 2.3. Carbon Monoxide Decreased Apoptosis of AKI in Septic Rats

**Figure 3:**
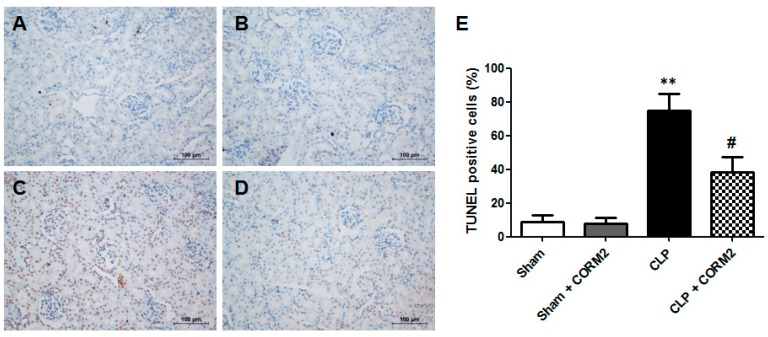
Carbon monoxide significantly decreased apoptosis in renal tissues of septic rats. (**A**–**D**) The renal tissues apoptotic analysis was performed by TUNEL staining; (**A**) Sham group; (**B**) Sham + CORM-2 group; (**C**) CLP group; (**D**) CLP + CORM-2 group. Scale bar = 100 μm; (**E**) Semi-quantitative apoptotic cells (TUNEL positive cells, brown cells in **A**–**D**) in renal tissues of septic rats. CLP-induced sepsis lead to increase of apoptosis in renal tissues and CORM-2 treatment decreased apoptosis in renal tissues. CORM-2 had no effect on apoptosis in renal tissues to normal rats (Sham). Results are presented as mean ± SD, *n* = 6 rats per group. ** *p* < 0.01 *vs.* sham group and **^#^** *p* < 0.05 *vs.* CLP group.

The terminal deoxynucleotidyl transferase-mediated dUTP-biotin nick end labeling (TUNEL) assay was performed to detect the apoptotic cells in renal tissues at 24 h after CLP surgery. The number of TUNEL-positive cells was significantly higher in the CLP group than in the sham-operated control or sham plus CORM-2 group. However, fewer TUNEL-positive cells were observed in the CLP plus CORM-2 group (Figure 3A–D). Quantification of TUNEL-positive cells in renal sections of the rats, the results showed that CORM-2 treatment significantly decreased the apoptotic cells of the CLP-induced AKI in rats (Figure 3E).

### 2.4. Carbon Monoxide Decreased Oxidative Damage of AKI in Septic Rats

**Figure 4:**
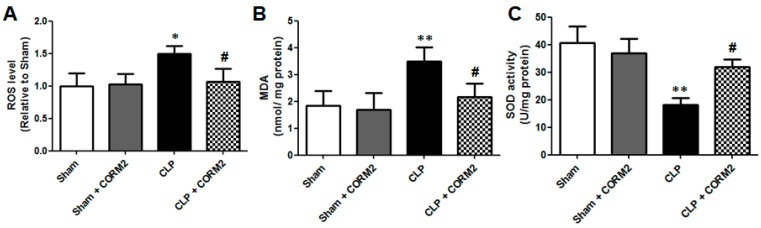
Carbon monoxide significantly decreased oxidative damage in renal tissues of septic rats. (**A**) CORM-2 treatment decreased CLP-induced ROS levels in renal tissues of septic rats; (**B**) CORM-2 treatment decreased CLP-induced MDA levels in renal tissues of septic rats; (**C**) CORM-2 treatment increased SOD activity in renal tissues of CLP-induced septic rats; CORM-2 had no effect on oxidative damage in renal tissues to normal rats (Sham). Results are presented as mean ± SD, *n* = 6 rats per group. * *p* < 0.05, ** *p* < 0.01 *vs.* sham group and **^#^** *p* < 0.05 *vs.* CLP group.

Oxidative stress is a predominant component involved in the pathogenesis of AKI [19]. Therefore, the reactive oxygen species (ROS) of the renal tissues were detected at 12 h after CLP surgery. As shown in Figure 4A, the ROS levels were significantly higher in the CLP group than in the sham-operated control or sham plus CORM-2 group (*p* < 0.01). However, CORM-2 treatment significantly decreased the ROS levels in CLP group (*p* < 0.05). Malondialdehyde (MDA) is one of the final products of ROS attack in phospholipids and is responsible for cell membrane damage. Thus, the MDA levels of the renal tissues were detected at 12 h after CLP surgery. The results showed that the MDA levels were significantly higher in the CLP group than in the sham-operated control or sham plus CORM-2 group (*p* < 0.01), whereas CORM-2 treatment significantly decreased the MDA levels in CLP group (*p* < 0.05) (Figure 4B). The superoxide dismutase (SOD) activity was measured to test the effect of CORM-2 on antioxidative ability in renal tissues at 12 h after CLP surgery. The results showed that the SOD activity was significantly decreased in the CLP group than in the sham-operated control or sham plus CORM-2 group (*p* < 0.01), while CORM-2 treatment significantly increased the SOD activity in CLP group (*p* < 0.05) (Figure 4C). Taken together, these results suggested that carbon monoxide decreases oxidative damage of AKI in septic rats.

### 2.5. Carbon Monoxide Decreased TNF-α and IL-1β Levels of AKI in Septic Rats

**Figure 5:**
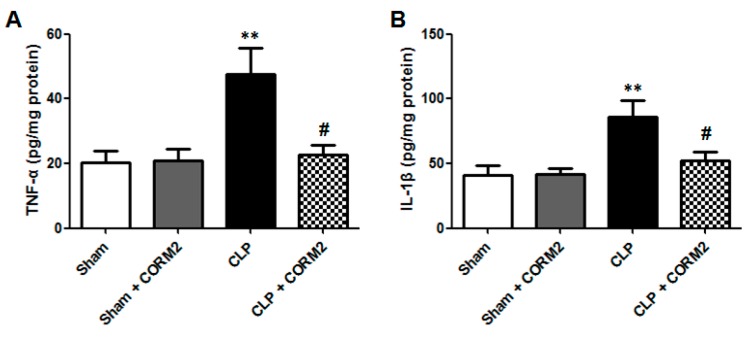
Carbon monoxide significantly decreased TNF-α and IL-1β levels in renal tissues of septic rats. (**A**) The TNF-α and (**B**) IL-1β levels in renal tissues were significantly increased at 12 h after CLP surgery, but CORM-2 treatment significantly decreased the TNF-α level in renal tissues. CORM-2 had no effect on TNF-α or IL-1β levels in renal tissues to normal rats (Sham). Results are presented as mean ± SD, *n* = 6 rats per group. ** *p* < 0.01 *vs.* sham group and **^#^** *p* < 0.05 *vs.* CLP group.

ROS can directly or indirectly elevate inflammation, and trigger the expression of inflammatory cytokines [20], so the TNF-α and IL-1β levels in renal tissues were measured at 12 h after CLP surgery. The TNF-α (Figure 5A) and IL-1β (Figure 5B) levels were significantly increased in the CLP group compared to the sham-operated control or sham plus CORM-2 group (*p* < 0.01). However, CORM-2 treatment significantly decreased the ROS levels in CLP group (*p* < 0.05). Together, these data suggested that CORM-2 treatment reduces the release of cytokines in CLP-induced AKI.

### 2.6. Carbon Monoxide Inhibited NLRP3 Inflammasome Activation of AKI in Septic Rats

**Figure 6:**
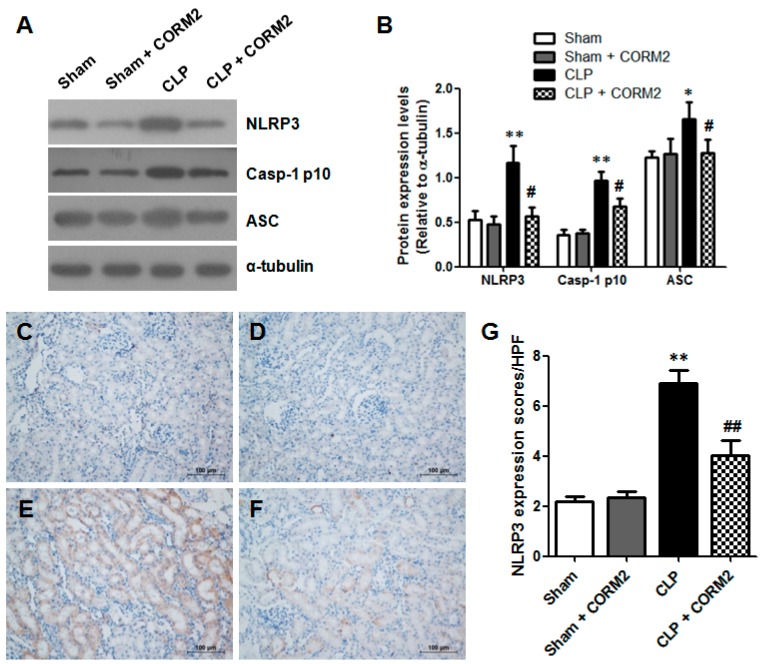
Carbon monoxide decreased NLRP3 inflammasome activation in renal tissues of septic rats. (**A**) NLRP3, Caspase-1 p10 (Casp-1 p10) and ASC protein expressions in renal tissues were determined by western blot. α-tubulin was measured as the loading control; (**B**) Quantification of the protein expressions by measuring bands intensity in western blot. CLP-induced sepsis lead to increase of NLRP3 and Caspase-1 p10 expressions in renal tissues, but CORM-2 treatment decreased NLRP3 and Caspase-1 p10 expressions in CLP-induced renal tissues. CORM-2 had no effect on NLRP3 inflammasome activation in renal tissues to normal rats (Sham). Results are presented as mean ± SD, *n* = 3 rats per group. * *p* < 0.05, ** *p* < 0.01 *vs.* sham group and **^#^** *p* < 0.05 *vs.* CLP group; (**C**–**F**) The NLRP3 expression was evaluated in renal tissues by immunohistochemical staining; (**C**) Sham group; (**D**) Sham + CORM-2 group; (**E**) CLP group; (**F**) CLP + CORM-2 group. Scale bar = 100 μm; (**G**) Semi-quantitative the NLRP3 expression (brown cells in **C**–**F**) in renal tissues of septic rats. CLP-induced sepsis lead to increase of NLRP3 expression in renal tissues, but CORM-2 treatment decreased NLRP3 expression in CLP-induced renal tissues. CORM-2 had no effect on NLRP3 expression in renal tissues to normal rats (Sham). Results are presented as mean ± SD, *n* = 6 rats per group. ** *p* < 0.01 *vs.* sham group and **^##^** *p* < 0.01 *vs.* CLP group.

Since IL-1β is a NLRP3 inflammasome-dependent cytokine, so we detected the NLRP3 inflammasome expression and the effect of CORM-2 on NLRP3 inflammasome activation in renal tissues at 24 h after CLP surgery. The NLRP3, ASC, and caspase-1 p10 protein levels in renal tissues were measured by western blot (Figure 6A). The results showed that CLP increased the NLRP3 (*p* < 0.01), ASC (*p* < 0.05), and caspase-1 (*p* < 0.01) protein expression compared with the sham-operated control or sham plus CORM-2 group rats, while CORM-2 treatment significantly decreased these protein expressions compared to CLP group (*p* < 0.05) (Figure 6B). The NLRP3 expression was also examined by immunohistochemical staining in renal tissues at 24 h after CLP surgery (Figure 6C–F). As shown in Figure 6G, The NLRP3 expression was significantly higher in the CLP group than in the sham-operated control or sham plus CORM-2 group (*p* < 0.01). However, CORM-2 treatment significantly decreased the NLRP3 expression compared to CLP group (*p* < 0.01). Together, these data suggested that CORM-2 treatment inhibit the NLRP3 inflammasome activation in sepsis-induced AKI.

## 3. Discussion

AKI is a frequent and severe complication of sepsis in intensive care unit (ICU) patients. Additionally, there is strong evidence that sepsis and septic shock are the most important causes of AKI in critically ill patients, which account for 50% or more of cases of AKI in ICUs [1,2]. CLP surgery may induce the activation of an inflammatory cascade, leading to sepsis and multiple organ failure [21]. CLP animal model has been widely used in sepsis and AKI studies [2]. In the present study, the CLP rat model was used to evaluate the possible role of exogenous CO liberated from CORM-2 in sepsis-induced AKI. The significant increase of BUN, Scr levels, and renal histopathologic changes after CLP surgery indicated obvious renal function injury. However, our results demonstrated that CORM-2 treatment significantly decreased sepsis-induced AKI and improved the survival of the septic rats.

Oxidative stress is a key component involved in the pathogenesis of AKI, and it occurs when the generation of pro-oxidants or ROS exceeds endogenous antioxidant capacity [22,23]. ROS are the main oxidants to oxidize cellular biomolecules and ultimately lead to cellular demise. Antioxidant enzymes such as superoxide dismutase and catalase have evolved as cellular defenses to ROS [24]. Mitochondrial respiratory chains and NADPH oxidase are the major common sources of ROS [24]. The kidney tissues are highly vulnerable to damage caused by ROS, because mitochondria are rich in kidney organ [19,25]. In our study, the ROS and MDA levels were significantly increased after CLP surgery in renal tissues, but CORM-2 treatment decreased the ROS levels and increased the SOD activity. Therefore, our results suggest that CO can decrease the oxidative stress by increase of SOD activity in sepsis-induced AKI. CO can directly inhibit mitochondria respiration and NADPH oxidase, so it may lead to a decrease in cell ROS levels [26,27]. It has been reported that exogenous CO induced NRF2 activation and the prevention of oxidative stress [28]. NRF2 can coordinately upregulate expression of several antioxidative enzymes by binding to antioxidant response elements of antioxidant genes to increase their transcription, including SOD, and glutathione S-transferase, as well as NAD(P)H:quinone oxidoreductase 1 [29].

Oxidative stress is well known to induce inflammation and both processes are deeply interrelated in sepsis and AKI [19]. ROS directly or indirectly triggers inflammation by increasing the expression of pro-inflammatory cytokines and chemokines, and it has also been suggested as an activator of the NLRP3 inflammasome [5,30]. Therefore, the inflammation and expression of NLRP3 inflammasome was detected in sepsis-induced AKI. Our data showed that the TNF-α and IL-1β levels were significantly increased in renal tissues at 12 h after CLP surgery, and NLRP3 inflammasome protein expressions were elevated at 24 h after CLP surgery. CO can decrease inflammatory cytokines expression by inhibiting NF-κB activation and the expression of granulocyte-macrophage colony stimulating factor, which enhance the secretion of inflammatory cytokines including TNF-α [31,32]. Acute tubular necrosis is the most common cause of acute renal injury, and it involves in an inflammatory response that release of cytokines to the kidney, which is detrimental and worsens renal injury [33]. The role of the NLRP3 inflammasome in AKI is further demonstrated by the finding that caspase-1-deficient mice are more resistant to acute tubular injury in cisplatin or ischemia-induced acute renal failure [7,34].

The anti-inflammatory action of CO has been demonstrated in several animal models, suggesting it can be used a therapeutic drug for inflammatory diseases. For example, systemic administration of CORM-2 in septic mice attenuates the accumulation of neutrophils, the expression of intracellular adhesion molecule 1, and the activation of B cells [32]. In our study, CO inhibited the NLRP3 inflammasome activation and decreased the sepsis-induced AKI in rats. Previous studies suggested that NLRP3 inflammasome activity is positively regulated by ROS [5,30], so CO may inhibit NLRP3 inflammasome activation by decreasing ROS levels in kidney of sepsis-induced AKI. CO can activate nitric oxide (NO) synthase and increase the levels of NO, which has many roles in immune responses [35]. It has been demonstrated that NO negatively regulated the activation of the NLRP3 inflammasome by stabilizing mitochondria in macrophages and prevented lipopolysaccharide-induced septic shock [36]. Therefore, CO may also inhibit NLRP3 inflammasome activation by increasing NO levels in sepsis-induced AKI.

## 4. Experimental Section

### 4.1. Reagents

Tricarbonyldichlororuthenium(II) dimer ((Ru(CO)_3_Cl_2_)_2_ or CORM-2) was obtained from Sigma Aldrich (St. Louis, MO, USA) and was dissolved in dimethyl sulfoxide (DMSO) as a stock solution. Pentobarbital sodium was purchased from Sigma Aldrich. Rat tumor necrosis factor alpha (TNF-α) and interleukin-1β (IL-1β) enzyme-linked immunosorbent assay (ELISA) kits were purchased from Cusabio (Wuhan, China). BUN and Scr detection kits were purchased from Nanjing Jiancheng Bioengineering Institute (Nanjing, China).The ROS detection kit was purchased from Genmed Scientifics (Shanghai, China). The MDA and SOD activity detection kits were purchased from Beyotime Biotechnology (Jiangsu, China). The bicinchoninic acid (BCA) protein assay kit was purchased from Pierce (Rockford, IL, USA). Anti-NLRP3, anti-ASC, anti-Caspase1 p10 and anti-tubulin antibody were purchased from Santa Cruz Biotechnology Inc. (Santa Cruz, CA, USA). The TUNEL assay kit was purchased from Roche (Mannheim, Baden-Württemberg, Germany).

### 4.2. Animals

Male Sprague-Dawley rats (body weight, 190–210 g) were purchased from the Medical Experimental Animal Center of Guangdong Province (Guangzhou, China). Animals were maintained on laboratory chow and housed in a specific pathogen-free room at constant temperature (20–22 °C) with 10 h of light and 14 h of dark exposure. All of the animal studies were performed in accordance with the Guidelines for the Care and Use of Laboratory Animals of the National Institutes of Health and were approved by the Institutional Animal Care and Use Committee of Sun Yat-sen University (NO. SCXK2011-07-0029).

### 4.3. Animal Model of AKI and Treatment

The animal model of sepsis induced AKI was performed through CLP surgery as previously described with minor modifications [37]. Briefly, rats were anesthetized with sodium pentobarbital (50 mg/kg) given intraperitoneally. The cecum was tightly ligated with 3-0 silk suture at half the distance between the distal pole and the base of cecum and perforated twice with a 22-gauge needle. A small amount of feces was extruded from both the mesenteric and antimesenteric penetration holes to ensure patency. Then, the cecum was returned to the peritoneal cavity without removing the cecal ligature, and the incision was closed with 4-0 surgical sutures.

After CLP surgery, the animals were randomly divided into four groups for treatment: sham group (sham), sham plus CORM-2 group (sham + CORM-2), CLP group (CLP) and CLP plus CORM-2 group (CLP + CORM-2). In the sham group, rats underwent the same surgical procedures, but the cecum was neither ligated nor perforated. CORM-2 stock solution was dissolved in normal saline and injected into animals intraperitoneally at 10 mg/kg in sham + CORM-2 group and CLP + CORM-2 group 3 h after surgery. Rats in sham group and CLP group received equal volumes of intraperitoneal injections of normal saline at the corresponding time points.

### 4.4. Measurement of Scr and BUN (Blood Urea Nitrogen)

Blood samples were collected at 12 h after CLP surgery in each group and renal function was monitored by measuring the concentration of BUN and Scr in the serum using detection kits (Nanjing Jiancheng, Nanjing, China) according to the manufacturer’s instruction.

### 4.5. Cytokine Assays

The renal tissues were harvested and homogenized in phosphate buffer at 12 h after CLP surgery in each group. The concentrations of TNF-α and IL-1β in the renal tissues were measured by ELISA kit (Cusabio, Wuhan, China) according to the manufacturer’s instruction. Cytokine concentration was then normalized by tissues protein concentration.

### 4.6. Measurement of ROS (Reactive Oxygen Species), MDA (Malondialdehyde) Levels and SOD (Superoxide Dismutase) Activity

The renal tissues were harvested and homogenized in phosphate buffer at 12 h after CLP surgery in each group. The ROS (Genmed Scientifics, Shanghai, China), MDA levels and SOD activity (Beyotime, Nantong, China) were measured according to the manufacturer’s instruction. The MDA levels and SOD activity were then normalized by tissues protein concentration.

### 4.7. Western Blotting Analysis

The renal tissues were homogenized in a RIPA lysis buffer at 24 h after CLP surgery in each group, and the lysates were cleared by centrifugation (14,000 rpm) at 4 °C for 30 min. Approximately 40 μg protein of each sample were run on a 10% SDS-PAGE gel and transferred to PVDF membranes. After blocking the non-specific binding sites for 60 min with 5% non-fat milk, the membranes were incubated with primary monoclonal antibodies against NLRP3 (Santa Cruz Biotechnology, Santa Cruz, CA, USA, 1:1000 dilution), ASC (Santa Cruz Biotechnology, 1:1000 dilution), Caspase1 p10 (Santa Cruz Biotechnology, 1:1000 dilution) and α-tubulin (Santa Cruz Biotechnology, 1:2000 dilution) overnight at 4 °C. Next, the membranes were subjected to three 15 min washes with TBST and then incubated with HRP-conjugated secondary antibody (at a 1:2000 dilution) for 45 min at room temperature. The membrane was washed three more times with TBST and developed using an enhanced chemiluminescence system (Cell Signaling Technologies, Beverly, MA, USA). The relative optical densities of specific proteins were determined using a Syngene G:BOX analysis program (Syngene, Cambridge, UK).

### 4.8. Histological Examination Analysis

The renal tissues were harvested at 24 h after CLP surgery in each group. The tissues were immediately fixed in 10% formaldehyde and then paraffin-embedded and sliced. The prepared tissue samples were stained with hematoxylin and eosin (HE) and observed under a light microscope for histological analysis. Pathological changes of the renal tissues were reviewed blindly and scored using a semiquantitative scoring system previously described with minor modifications [38]. Specifically, for each kidney 50 cortical tubules from at least 10 different fields were scored and the mean scores per field in the same group were calculated. Higher scores represented more severe damage (the maximum score per field was 10).

### 4.9. Immunohistochemistry and TUNEL Assay

The renal tissues were harvested at 24 h after CLP surgery in each group. The tissues were immediately fixed in 10% formaldehyde and then paraffin-embedded and sliced. The slides were deparaffined and blocked endogenous peroxidase activity by incubation in 3% hydrogen peroxidase. To retrieve the antigenicity, the slides were immersed in EDTA (1 mmol/L, pH 8.0) and boiled for 15 min in a microwave oven. The sections were then incubated with a primary antibody against NLRP3 (Santa Cruz, USA, 1:100 dilution) at 4 °C overnight, washed, and then incubated with secondary antibody at room temperature for 30 min. 3,3′-diaminobenzidine tetrahydrochloride (DAB) was used for signal development after washing with PBS three times, and then the sections were counterstained with 20% hematoxylin. The immunostaining score was calculated as a percentage of positively stained cells multiplied by the staining intensity. The percent was positively scored as 1 (5%–25%), 2 (25%–50%), and 3 (>50%). The staining intensity was scored as 0 (no staining), 1 (weakly stained), 2 (moderately stained), and 3 (strongly stained).

The TUNEL assay was performed using the *in situ* Cell Death Detection kit (Roche, Mannheim, Baden-Württemberg, Germany) according to the manufacturer’s instructions. Seven fields of vision (magnification of 40×) in the renal sections were randomly sampled from each kidney, and positive staining of cell nuclei (brown) was identified under a light microscope.

### 4.10. Statistical Analysis

All measurement data were presented as mean ± standard deviation (SD), and the statistical analyses were performed using SPSS version 19.0 for Windows. Unpaired t test was used to compare the same parameters between two groups. Comparison of the same parameters among three groups was done using one-way analysis of variance (ANOVA), and the difference between pairs of means was tested post hoc with Tukey’s test. The difference in survival rates among the groups was compared using Kaplan-Meier survival analysis. *p* values of less than 0.05 were considered statistically significant.

## 5. Conclusions

The present study provides a basis for the use of CORM-2 as an effective strategy to protect against sepsis-induced AKI, and exogenous CO as released by CORM-2 treatment decreases the oxidative stress and NLRP3 inflammasome activation. However, the mechanisms of CO and NLRP3 inflammasome inhibition remain to be clarified. In addition, the potential beneficial effect of exogenous CO may highly depend on the pathological condition, the mode, duration of application, and the administered concentration. Further studies are required to clarify the pharmacokinetics and biology of CO and CORM-2.
